# APOE-ɛ4 effects on longitudinal decline in olfactory and non-olfactory cognitive abilities in middle-aged and old adults

**DOI:** 10.1038/s41598-017-01508-7

**Published:** 2017-04-28

**Authors:** Maria Josefsson, Maria Larsson, Steven Nordin, Rolf Adolfsson, Jonas Olofsson

**Affiliations:** 1 Centre for Demographic and Ageing Research, Umeå University, Umeå, Sweden; 2 Gösta Ekman Laboratory, Department of Psychology, Stockholm University, Stockholm, Sweden; 3 Department of Psychology, Umeå University, Umeå, Sweden; 4 Department of Clinical Sciences, Psychiatry, Umeå University Hospital, Umeå, Sweden; 5 Swedish Collegium for Advanced Study, Uppsala, Sweden

## Abstract

Characterizing aging-related decline trajectories in mental abilities, and relationships of the ɛ4 allele of the Apolipoprotein gene, helps to identify individuals at high risk for dementia. However, longitudinal changes in olfactory and non-olfactory cognitive abilities have not been investigated in relation to the ɛ4 allele. In the present study, participants from a large population-based study (657 middle-aged and 556 old) were tested over 10 years on their performance on an odor identification task and three non-olfactory cognitive tasks; MMSE, episodic memory, and semantic memory. Our key finding is that in middle-aged participants, odor identification declined twice as fast for ɛ4/4 homozygotes, compared to non-carriers. However, in old participants, the ɛ4/4 homozygotes showed an impaired odor identification ability, but they declined at a similar rate as the non-carriers. Furthermore, in old participants all assessments displayed aging-related declines, but exaggerated declines in ɛ4-carriers were found only in MMSE and episodic memory assessments. In sum, we present evidence that odor identification ability starts to decline already in middle-aged, and that carriers of ɛ4/4, who are at highest risk of developing dementia, decline twice as fast. Our results may have implications for use of odor identification assessment in detection of early-stage dementia.

## Introduction

Deficits in olfactory functioning are characteristic of age-related disorders such as mild cognitive impairment and Alzheimer’s disease (AD)^1, 2^. Olfactory impairment predicts future decline in several cognitive domains in older adults without dementia^3–5^, suggesting that olfactory dysfunction might provide an early marker for age-related cognitive impairment. However, little is known about how age-related olfactory dysfunction develops over time and how such decline is related to decline in other non-olfactory cognitive domains. Most evidence regarding effects of age on olfactory functions is based on cross-sectional observations, but such data is confounded by cohort effects and might not accurately reflect effects of aging^6^. Knowledge is thus warranted regarding age-related longitudinal olfactory change and its relationship to early-stage dementia and age-related changes in other cognitive domains^7, 8^.

Research on the relationship between olfactory and non-olfactory cognitive impairments has yielded mixed evidence. Some studies indicate that odor identification performance is a reliable predictor of subsequent decline in verbal episodic memory and processing speed abilities over a 6-year period^4, 9^. In contrast, Finkel *et al*.^10^ reported that odor identification ability was associated with verbal, episodic memory and processing speed abilities at baseline, but that baseline performance in odor identification was unrelated to the rate of decline in all of the non-olfactory cognitive functions over a 15-year period. Odor identification performance was also found to predict cognitive impairment, defined as a score of less than 24 points on the Mini-Mental State Examination (MMSE) over a 5-year period^11^. Two recent longitudinal studies involving 107 middle-aged and older participants who were followed across 6.5 years showed age-related decline in odor identification, episodic verbal memory, processing speed, executive functioning and language proficiency^12, 13^. Odor identification ability was a significant predictor of episodic memory outcomes. In a follow-up study, Wehling *et al*.^13^ reported high correlations among four different olfactory tasks (i.e., uncued odor identification, cued odor identification, perceived familiarity, perceived edibility) and a parallel age-related decline across all four tasks^13^. Overall, there is thus evidence to suggest that olfactory impairments are linked to impaired (non-olfactory) global cognitive function and fluid cognitive abilities.

Age-related olfactory impairment is influenced by genetic factors. From a cognitive aging perspective, the Apolipoprotein E (APOE) ɛ4 allele is of particular interest, as it is the most well-established genetic risk factor for AD^14^. The presence of the APOE ε4 allele is associated with AD-related pathology, prevalence of MCI, and accelerated cognitive decline^15–17^. Moreover, there is evidence of a dose dependent effect, such that carriers of two copies of the ε4 allele (ε4/4 homozygotes), compared to only one (ε4 heterozygotes), demonstrate higher risk for developing AD and a more rapid cognitive decline^18, 19^. Intriguingly, the APOE gene is also expressed in olfactory brain structures, including the olfactory bulb (OB) and the olfactory epithelium, and has been associated with a regeneration processes that occurs in the olfactory nerve^20^. Results show an association between olfactory impairment and presence of the ɛ4 allele^21–23^, and more pronounced olfactory impairments among ε4/4 homozygous older adults diagnosed with probable AD^24^. Previous work from our group indicates that APOE-ɛ4 carriers with olfactory dysfunction at baseline display a prospective MMSE decline (5 years) that is about twice as large as that observed either in non-carriers, or in ɛ4 carriers with normal olfactory function^22^. We recently reported that ɛ4-carriers with an exaggerated episodic memory decline over a 10-year time period also exhibited marked olfactory deficits at follow-up. This was in sharp contrast to those with memory decline but no ɛ4, who showed no olfactory impairment^5^.

Given that the overall pattern of findings suggest that the APOE-ɛ4 is an important moderator of age-related olfactory and cognitive impairments, the present study was designed to investigate the role of ɛ4 in olfactory and (non-olfactory) cognitive trajectories across a 10-year interval using observations from a large, prospective, and population-based sample (n ~ 1000). We extend previous work, first, by comparing, for the first time, aging-related decline slopes of odor identification and non-olfactory cognitive tests, and second, by investigate how the APOE-ɛ4, the most prominent genetic risk factor for late-onset AD, influences the longitudinal trajectories of these assessments. The specific questions examined were 1) Is there a dose-dependent association between ɛ4 and 10-year change in olfactory and (non-olfactory) cognitive trajectories? 2) Is the effect, if any, established already in middle age? 3) Are olfactory and non-olfactory cognitive assessments characterized by similar longitudinal change patterns? We hypothesized that odor identification would show a decline at early age, and that there would be a dose dependent effect of ɛ4 related to steeper decline across age in both olfactory and non-olfactory functions. We also hypothesized that odor identification decline would show a pattern similar to episodic memory in ɛ4 carriers, as they both are assumed to rely on mediotemporal lobe structures affected by the APOE.

## Materials and Methods

### Design

The Betula Project is a population-based prospective study that was initiated in 1988 with focus on aging, memory, and health^25^. Participants considered for inclusion in the present study were recruited at the first and second wave of data collection (T1: 1988–1990 and T2: 1993–1995). At recruitment, the samples comprised 1000 individuals each, evenly distributed over 10 age cohorts ranging from 35 to 85 years of age and approximately even gender distributions (full sample characteristics are provided in ref. 25). Participants were assessed every five years, and at each test wave assessment was conducted of health and cognitive performance. The study was conducted in Umeå, a city of about 125,000 inhabitants located in northern Sweden. The participants’ written consent was obtained for the study in accordance with the Declaration of Helsinki and the study was approved by the Regional Ethical Vetting Board at Umeå University (approval no. 870303, 97–173, 221/97, 97–173, 03–484, 01–008, 169/02, 02–164, 03–484, 05-082 M, and 08-132 M).

### Odor Identification

During the third test wave, T3,1998–2000 a modified version^26^ of the Scandinavian Odor Identification Test^27^ was included to assess odor identification ability. The assessment consisted of thirteen household odor stimuli that are familiar to the Scandinavian population. For each stimulus, participants are provided with a written list of four alternatives, of which one is the correct source label. Odor identification performance was determined as the number of correctly identified odors (range 0–13), in which a higher score represents better performance.

### Non-Olfactory Cognitive Measures

Three assessments were used to characterize non-olfactory cognitive function, and these assessments were included in all test waves in the Betula study. The first assessment was the Synonym Reasoning Blocks (SRB), a forced-choice vocabulary test including 30 words for which participants were instructed to find synonyms from four written alternatives^28^. The test format is similar to the odor identification test and both tasks draw on semantic knowledge^29^. The second assessment was the MMSE, which is commonly used to assess general cognitive dysfunction^30^. The third assessment was a composite episodic memory score (EMS) based on the sum of scores on five different episodic memory tasks. The episodic memory tasks were: (1) immediate free recall of 16 visually and orally presented short sentences, (2) delayed cued recall of nouns from the previously presented sentences, (3) immediate free recall of 16 enacted sentences, (4) delayed cued recall of nouns from the enacted sentences, and (5) immediate free recall of a list of 12 orally presented nouns. Two different sets of sentences and nouns were used alternately in the first four measures, and four sets were used alternately in the fifth measure^17^. The EMS score could range from 0 (minimum) to 76 (maximum).

### APOE Genotyping

A polymerase chain reaction (PCR) was performed with 200 ng genomic DNA as template in a 25:1 reaction mixture containing 20 pmol of PCR primers APOE-A (5′-TCCAAGGAGCTGCAGGCGGCA-3′) and APOE-B: (5′-ACAGAATTCGCCCCGGCCTGGTACACTGCCA-3′^31^; 0.2 units (U) Taq DNA polymerase (Gibco BRL, Gaithersburg, MD); 1.0 mM MgCl_2_; 75 mM Tris-HCI pH 9.0; 20 mM (NH_4_)_2_SO_4_; and 10% DMSO. The PCR amplification consisted of 35 cycles of 30 s at 94 °C, 30 s at 72 °C, PCR products were digested with 5 units HhaI (Life Technologies, Portland, OR) by incubating for 3 hr at 37 °C. Bands were separated on a 5% agarose gel and visualized on an ultraviolet transilluminator after ethidium bromide staining. Alternatively, electrophoresis was performed with ExcellGel-gels (Pharmacia, acquired by Pfizer, New York) and the MultiphorII electrophoresis system (Pharmacia/Pfizer), and the bands were visualized by silver staining.

### Dementia assessment

Dementia diagnoses were obtained through comprehensive evaluation of medical records to identify symptoms indicative of progressive dementia. The evaluation was coordinated by the same senior research geropsychiatrist. Following a procedure in which participants fulfilling one or several of the following criteria were considered “at higher risk” and were more extensively evaluated: (a) suspected dementia signs observed by the staff conducting the health assessments and cognitive testing, (b) MMSE performance below 24, (c) a decline in the MMSE score (at least three points) from the previous testing occasion, or (d) a subjective sense of memory impairment reported by the participant. Diagnoses were based on the DSM-IV criteria^32^.

### Statistical Analysis

To assess differences in longitudinal performanceon olfactory and cognitive tasks across APOE genotypes we considered non-carriers of ɛ4 as the reference group in all analyses. The Student t-test and Fisher’s exact test were used to compare demographic measures among the three APOE groups (corresponding to 0, 1 and 2 alleles of the ɛ4). As our prior results showed no effect on odor identification performance for the ɛ2 allele relative to the most common ɛ3 allele, only ɛ4 was considered^33^.

We used linear mixed models^34^ to analyze the association between the APOE gene and 10-year change in olfactory and non-olfactory cognitive performance. To compare results across tests, the outcomes were transformed to percentages. Fixed-effect terms included an overall intercept term, binary indicator variables for ɛ4/4 homozygotes and ɛ4 heterozygotes, plus interactions with time. Time was considered as a continuous covariate, measured in decades from baseline (such that the parameter estimate corresponds to a 10-year change). The model also included covariate characteristics previously shown in the literature to be associated with the outcomes; age, sex, education, and an indicator variable to account for potential effects related to testing experience prior to the first olfactory assessment (one vs. two test occasions). All covariates were included as main effects and as interactions with time. Further, the model included a subject-specific random intercept to account for within-subject correlations between repeated measurements. Continuous covariates were standardized to a mean of zero and a variance of one to ease interpretation of parameter estimates and reduce multicollinearity^35^. Missing covariates (Education; n = 7) were imputed with the age-specific mean score.

Although the study was based on random samples from the population at enrollment, attrition during the 10-year period leading up to the third wave (when the SOIT was included) was substantial (30%), as can be expected given the duration of the study and age of participants. To adjust for possible selection bias due to attrition in the statistical analysis we used inverse probability weighting^36^ (IPW). It is based on assigning a weight to each subject participating at the third wave so that the subject accounts in the analysis not only for themself, but also for those with similar characteristics who dropped out. The individual weight is the inverse estimated probability of participating at the third wave. The weights were estimated using logistic regression models conditioning on APOE genotype, covariates described above, and cognitive measures at enrollment. The outcome of the model was whether or not the subject participated in the olfactory and non-olfactory cognitive testing at the third test wave. Attrition is not assumed to be random with respect to the cognitive outcomes or APOE genotype. As such, unadjusted analyses would underestimate cognitive and olfactory decline and/or underestimate genotype differences. Moreover, since IPW can only yield unbiased results if there are no unmeasured confounders^36^, we conducted a sensitivity analysis to explore the sensitivity of the estimates in the longitudinal models to the unmeasured odor identification task at enrollment when estimating the IPW model. We followed the simulation procedure reported in prior research^37^, assuming a moderate effect, comparable to the parameter estimates of the other non-olfactory cognitive tasks, of odor identification on the probability of participating in the cognitive testing at the third wave. Details are given in the supplementary material.

We further used partial correlations to measure the association between baseline intercepts and rates-of-change between the olfactory and non-olfactory cognitive tasks, after controlling for demographic covariates. As a first step, for each subject and task, we calculated the intercept and rate-of-change using a linear regression where time was an exploratory variable. Moreover, to calculate the partial correlation between baseline intercepts or rates-of-change for two tasks, we first solved linear regressions with intercepts or rate-of-change as the outcome when controlling for demographic covariates and extracted the residuals. In a third step, we calculated the correlation between the residuals.

Statistical analyses were performed in R, version 3.2.2^38^ using the “lme4” package. P-values were calculated using Satterthwaite’s approximation^39^, which is implemented in the ‘lmerTest’ package.

### Study sample

The inclusion criteria for the present study were: (1) completing the olfactory and non-olfactory cognitive testing at the third test wave, (2) undergoing APOE genotyping, (3) not being diagnosed with dementia at the third wave, and (4) having a MMSE score higher than 24 at the third wave. In total, 1213 participants were included in the main analyses. Among the participants, 70.2% were without a ε4 allele (non-carriers), 27.6% were ε4 heterozygous, and 2.1% were ε4/4 homozygous, which is consistent with other studies^40^.

To investigate the combined effects of age and APOE genotype, the participants were divided into two age groups: middle-aged participants ranging from 45–60 years (n = 657), and old participants ranging from 65–85 years of age (n = 556). See Table 1 for the demographic and APOE data.

**Table 1:** Demographic characteristics stratified by APOE genotype.

	Middle aged			**Old**		
	*Non-carriers*	ε4	ε4/4	*Non-carriers*	ε4	ε4/4
N	450	190	17	402	145	9
% males	48	46	47	39	33	35
Education (yrs)	12.7 (3.5)	12.7 (3.7)	12.4 (3.6)	8.5 (3.3)	8.9 (3.6)	9.3 (2.6)
Age (yrs)	52.4 (5.7)	52.5 (5.5)	51.5 (6.8)	72.8 (6.5)	71.6 (6.4)	74.4 (6.4)

## Results

Pairwise t-tests and Fisher’s exact test revealed no significant differences in baseline demographic and cognitive characteristics comparing APOE ɛ4 carriers (ɛ4 heterozygotes or ɛ4/4 homozygotes) with non-carriers in this sample.

Among the old participants, results obtained from the logistic regression IPW models revealed a positive significant association between scores on the cognitive tests (MMSE, EMS, and SRB) at enrollment, and meeting the inclusion criteria for this study. Also, participants enrolled at the second wave, compared to those enrolled at the first wave, and younger participants, were more likely to participate at the third wave. Among the middle-aged participants, only time of enrollment was a significant predictor of participating in the third wave (see supplementary material for the results of the models).

**Figure 1:**
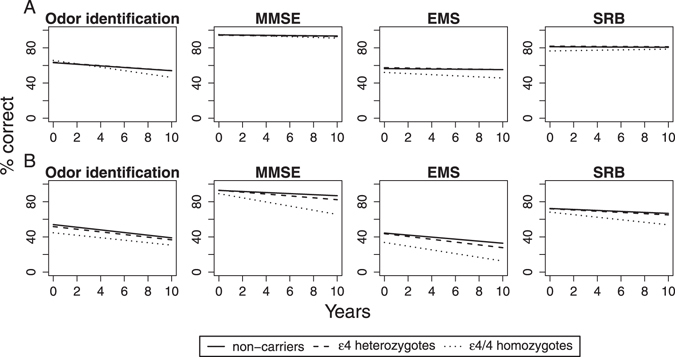
Estimated 10-year change in odor identification and non-olfactory cognitive tasks stratified by APOE group. Intercept and slopes are derived from mixed models. Panel A: Middle-aged participants. Panel B: Old participants.

Results of the weighted linear mixed models are given in Tables 2 and 3, and the corresponding olfactory and (non-olfactory) cognition trajectories over 10 years are plotted in Fig. 1. We specified separate models for old and middle-aged participants. After adjusting for demographic covariates, we compared baseline performance levels and rates of decline across APOE genotypes. In middle-aged participants, the results revealed no significant baseline differences in olfactory or non-olfactory cognitive tasks across APOE groups. However, there was an age-related decline in odor identification and MMSE scores. Furthermore, odor identification showed an exaggerated decline for ɛ4/4 homozygotes, compared to non-carriers. The results did not show a decline in SRB or in the EMS scores in middle-aged adults, although a borderline significant decline was shown for ɛ4/4 homozygotes in EMS, perhaps suggesting a similar decline pattern between EMS and odor identification.

**Table 2:** Parameter estimates from mixed models of olfactory and non-olfactory cognitive outcomes for middle-aged participants, adjusted for age, sex, education, and previous testing experience (one vs. two times).

	Odor identification		MMSE		EMS		SRB	
	Est (SE)	p-value	Est (SE)	p-value	Est (SE)	p-value	Est (SE)	p-value
Intercept	63.2 (1.03)	<0.001	94.8 (0.33)	<0.001	56.4 (0.81)	<0.001	81.2 (0.77)	<0.001
ε4	0.40 (1.24)	0.750	−0.47 (0.40)	0.243	1.05 (0.97)	0.276	0.64 (0.97)	0.484
ε4/4	2.56 (3.53)	0.468	0.34 (1.15)	0.766	−4.34 (2.76)	0.115	−4.77 (2.67)	0.069
10 Y change	−9.14 (1.43)	<0.001	−1.34 (0.47)	0.004	−1.15 (0.78)	0.142	−0.65 (0.62)	0.296
10 Y change × ε4	−0.30 (1.71)	0.863	−0.06 (0.56)	0.920	−1.05 (0.93)	0.259	−0.00 (0.74)	0.998
10 Y change × ε4/4	−10.20 (5.09)	0.045	−2.78 (1.63)	0.089	−5.22 (2.78)	0.061	2.73 (2.16)	0.210

**Table 3:** Parameter estimates from mixed models of olfactory and non-olfactory cognitive outcomes for old participants, adjusted for age, sex, education, and previous testing experience (one vs. two times).

	Odor identification		MMSE		EMS		SRB	
	Est (SE)	p-value	Est (SE)	p-value	Est (SE)	p-value	Est (SE)	p-value
Intercept	54.0 (1.20)	<0.001	92.8 (0.62)	<0.001	44.4 (0.98)	<0.001	72.3 (1.23)	<0.001
ε4	−2.25 (1.48)	0. 129	0.15 (0.77)	0. 844	−0.71 (1.22)	0. 562	−0.29 (1.53)	0. 848
ε4/4	−9.31 (4.77)	0. 052	−3.73 (2.43)	0.1266	−10.7 (4.09)	0. 009	−4.17 (5.17)	0. 420
10 Y change	−15.1 (1.82)	<0.001	−6.04 (0.98)	<0.001	−11.6 (1.15)	<0.001	−5.57 (1.25)	<0.001
10 Y change × ε4	0.01 (2.26)	0. 995	−4.66 (1.20)	<0.001	−4.39 (1.43)	0.002	−1.45 (1.50)	0.336
10 Y change × ε4/4	0.98 (8.05)	0. 903	−17.5 (4.40)	<0.001	−9.56 (6.01)	0.112	−8.82 (6.38)	0.167

For the old group, there were no baseline performance differences between APOE groups in MMSE or SRB. However, the old ɛ4/4 homozygotes scored lower than non-carriers in odor identification and EMS at baseline. Moreover, all four assessments displayed age-related declines, although a more rapid decline was observed for ɛ4 heterozygotes in MMSE and EMS, and for ε4/4 homozygotes in MMSE, compared to non-carriers. In the old group, odor identification decline was not associated with the ɛ4 allele.

A sensitivity analysis was conducted to estimate potential effects of the unmeasured odor identification ability at enrollment in the IPW models on the longitudinal estimates. Although a moderate effect was assumed, the longitudinal results were not affected by the inclusion of an unmeasured covariate, suggesting that our models were robust (see supplementary material for the results of the sensitivity analyses).

We wanted to understand whether the observed ɛ4-related decline patterns were associated across tasks, as this might elucidate whether olfactory and cognitive systems are jointly affected by the ɛ4. Thus, in additional analyses we explored partial correlations between baseline scores and between rates-of-changes in the olfactory and non-olfactory cognitive tasks, after controlling for age, gender and education in the overall sample. The results are shown in Tables 4 and 5. In baseline task performance, non-carriers and ɛ4 heterozygotes showed significant positive correlations between all of the non-olfactory cognitive tasks, and baseline odor identification was significantly correlated with SRB and EMS. For ɛ4/4 homozygotes only baseline MMSE was significantly associated with baseline EMS, although it should be noted that the small number of homozygotes might have resulted in low statistical power to detect significant effects.

**Table 4:** Change-change partial correlations for middle aged and old participants after controlling for demographic covariates; age, sex, education, and an indicator variable to account for potential effects related to previous testing experience (one vs. two times).

	Odor identification		MMSE		EMS	
	Correlation	p-value	Correlation	p-value	Correlation	p-value
*Non-carriers*						
**MMSE**	0.044	0.247				
**EMS**	−0.035	0.358	0.140	0.000		
**SRB**	0.102	0.007	0.185	0.000	0.060	0.111
*ε*4 *Heterozygotes*						
**MMSE**	−0.025	0.675				
**EMS**	−0.032	0.600	0.193	0.001		
**SRB**	0.057	0.350	0.222	0.000	0.254	0.000
*ε4*/*4 Homozygotes*						
**MMSE**	−0.095	0.683				
**EMS**	0.459	0.036	−0.013	0.955		
**SRB**	−0.211	0.358	−0.263	0.237	−0.103	0.648

**Table 5:** Baseline Intercept partial correlations for middle aged and old participants after controlling for demographic covariates; age, sex, education, and an indicator variable to account for potential effects related to previous testing experience (one vs. two times).

	Odor identification		MMSE		EMS	
	Correlation	p-value	Correlation	p-value	Correlation	p-value
*Non-carriers*						
**MMSE**	0.045	0.186				
**EMS**	0.078	0.023	0.283	0.000		
**SRB**	0.161	0.000	0.253	0.000	0.342	0.000
ε4 *Heterozygotes*						
**MMSE**	0.008	0.887				
**EMS**	0.132	0.016	0.228	0.000		
**SRB**	0.109	0.046	0.312	0.000	0.393	0.000
ε4/4 *Homozygotes*						
**MMSE**	0.005	0.982				
**EMS**	−0.084	0.682	0.555	0.003		
**SRB**	−0.104	0.615	0.153	0.455	0.268	0.186

Regarding longitudinal rates-of-change, the analysis of ɛ4 heterozygotes showed significant positive correlations between all of the non-olfactory cognitive tasks. However, odor identification change was not correlated with changes in any other cognitive test. Among non-carriers, the rate-of-change in odor identification correlated positively with change in SRB. The non-olfactory cognitive tasks showed a significant correlation between MMSE and EMS, and between MMSE and SRB, while the correlation between EMS and SRB did not reach significance. The results of the correlations among ɛ4/4 homozygotes across the tasks revealed only one significant positive association, that between odor identification and EMS.

Separate analyses for the two age groups showed similar results for both baseline intercepts and slopes, although the change-change correlations were more pronounced among old participants than middle-aged, presumably a function of more pronounced functional decline and variability in old participants.

## Discussion

What are the trajectories by which olfactory and non-olfactory abilities change across the adult lifespan? We investigated longitudinal changes in odor identification and non-olfactory cognitive abilities across three testing occasions spanning an interval of up to 10 years. We extend previous studies, first, by comparing, for the first time, aging-related decline slopes of odor identification and non-olfactory cognitive performance, and second, by investigating how the APOE-ɛ4, the most prominent genetic risk factor for late-onset dementia, influences the longitudinal trajectories of these assessments. Our large, age-varied sample allowed us to compare APOE-ɛ4 non-carriers to ɛ4 heterozygotes and ɛ4/4 homozygotes by investigating whether longitudinal changes in olfactory and non-olfactory cognitive tasks are dependent on the number of ɛ4 alleles.

This novel approach to study odor identification decline over a decade-long interval revealed a decline in odor identification already among middle-aged APOE ε4/4 homozygotes; their decline was twice as fast as the non-carriers and ɛ4 heterozygotes. Notably, among older individuals, these homozygotic ɛ4/4-carriers declined at a similar rate as non-carriers, even though they had an impaired odor identification ability, presumably as a result of the exaggerated decline in middle-age. This introduces the notion that ɛ4-effects of odor identification starts earlier than what is previously known. As APOE is the major genetic risk factor for dementia of the Alzheimer type, our results strengthen the notion that odor identification might be a marker of pre-clinical dementia in middle age.

Overall, ɛ4/4 homozygotes showed an accelerated aging development compared to ɛ4 heterozygotes and non-carriers. This pattern is consistent with previous observations^24, 41^, and it supports the hypothesis of an “additive dose effect” of the ɛ4 allele.

Notably, episodic memory is typically sensitive to aging^25^, and our results show that in middle-age, episodic memory decline is correlated with odor identification decline in ɛ4/4 homozygotes. This result indicates that odor identification and episodic memory are jointly affected by ɛ4, as indicated by prior work^5^. Our results suggest that odor identification and episodic memory provide complementary functional assessments of the mediotemporal lobes, which are vulnerable to atrophy in AD dementia patients with ɛ4^42^.

A combination of the ε4 allele and olfactory impairment was suggested to be useful in predicting cognitive decline^43^. Our current findings support this notion and suggest that the effect might be pronounced in ε4/4 homozygotes. However, further investigations with a larger sample size of ε4/4 homozygotes are warranted in order to elucidate this relationship.

Our findings fit well with a recent study that targeted specific ɛ4-effects on olfactory behavior and physiology^44^. This study revealed an ɛ4-driven shift of odor-evoked physiological dysfunction that affected the olfactory bulb in younger mice, but affected the piriform cortex in older mice. We speculate that olfaction might be uniquely affected in younger individuals, and that this deficit generalizes to cognitive functions in older ages. Future work should evaluate whether ɛ4-effects are specific to odor identification tasks^45^, or whether they generalize to olfactory threshold assessments^21^.

Surprisingly, our results showed that a decline was observed also for MMSE among middle-aged participants. The MMSE is often used as a screening task, typically not sensitive to subtle cognitive deficits^30^. The observed decline in MMSE could reflect a systematic bias caused by the baseline ceiling effects in the MMSE score, which is difficult to adjust for statistically^46^. In a longitudinal setting this implies that high performing participants, initially scoring at maximum on the MMSE, will, as a result, receive a stable (zero) or negative decline, compared to other tests (without ceiling effects) where the rate-of-change can move in either direction. As a result, the analyses may have overestimated the rate of decline in MMSE in middle-aged participants.

Analyses of change-change partial correlations revealed associations mainly among the non-olfactory cognitive tasks in ε4 heterozygotes and non-carriers, and this result was pronounced among older participants. These non-olfactory associations indicate that cognitive decline tend to covary across different domains, as has been discussed within the “common-cause” framework of cognitive aging^47^.

Moreover, we found that among ε4 non-carriers odor identification and SRB decline rates were correlated. This may reflect the fact that both the olfactory task and SRB draw on semantic memory^7, 48^, especially at high levels of performance in these tasks. However, it should be noted that non-olfactory assessments were more closely associated with each other than with odor identification. This pattern suggests that odor identification in most individuals presents a rather distinct age-related decline trajectory, as was suggested in recent work using factor-analytic methods^29^. Longitudinal data from the Betula and other large-scale studies will be useful in further characterizing the distinct role of olfaction in aging and early stages of dementia.

Although participants in the Betula study have a comparatively low drop-out rate, attrition was found to be substantial between enrollment and the baseline olfactory task at the third wave. The attrition effect was stronger in older, ε4 heterozygotes, cognitively lower-performing participants, whereas younger participants showed less strong effects, which is consistent with previous research^17, 49–51^. The present work corrected for attrition using IPW methods, thereby, limiting the bias associated with the healthy participant effect.

## Conclusion

Olfactory and cognitive abilities decline as a function of age and APOE ɛ4 status. An odor identification decline is present already in middle-age, and ɛ4/4 homozygotes, who are at high risk of developing dementia in old age, decline twice as fast. In older individuals, in contrast, ɛ4 -carriers decline at a similar rate as non-carriers. These findings suggest that odor identification impairment might provide an early indication of the risk of aging-related memory loss and dementia.

## Electronic supplementary material

Supplementary information
