# Supplementary material for: APOE-ɛ4 effects on longitudinal decline in olfactory and non-olfactory cognitive abilities in middle-aged and old adults
Source: Sci Rep. 2017 Apr 28;7:1286. doi: 10.1038/s41598-017-01508-7 (PMC5430873; doi:10.1038/s41598-017-01508-7)
Supplement: Supplementary file 1 — Supplementary information [file 41598_2017_1508_MOESM1_ESM.pdf]

Supplementary information

APOE-ε4 and longitudinal decline in olfactory and non-olfactory cognitive outcomes in middle-aged and old adults

Maria Josefsson, Maria Larsson, Steven Nordin, Rolf Adolfsson, Jonas Olofsson

Table S1: Parameter estimates from the logistic regression model of the middle-aged participants. We used the complete set of enrolled participants who completed APOE genotyping, non-carriers: n=530, ε 4 heterozygotes: n=223, ε 4/4 homozygotes: n=20. The outcome of the model is whether or not the participant completed the olfactory and cognitive testing at the third test wave.

	Estimate	Std. Error	p-value
**Intercept**	1.327	0.179	<0.001
**Test experience**	0.697	0.230	0.002
**Male**	0.152	0.208	0.464
**Education**	0.064	0.125	0.605
**MMSE**	0.068	0.104	0.517
**EMS**	0.116	0.122	0.342
**SRB**	0.201	0.116	0.085
**Age**	-0.046	0.122	0.703
**ε 4**	0.112	0.227	0.623
**ε 4/4**	0.155	0.652	0.813

Table S2: Parameter estimates from the logistic regression model of the old participants using the complete set of enrolled participants who completed APOE genotyping, non-carriers: n=663, ε 4 heterozygotes: n=268, ε 4/4 homozygotes: n=21. The outcome of the model is whether or not the participant completed the olfactory and cognitive testing at the third test wave.

	Estimate	Std. Error	p-value
**Intercept**	-0.085	0.141	0.545
**Test experience**	1.307	0.175	<0.001
**Male**	-0.009	0.156	0.954
**Education**	0.007	0.087	0.932
**MMSE**	0.371	0.093	<0.001
**EMS**	0.500	0.106	<0.001
**SRB**	0.207	0.098	0.034
**Age**	-0.683	0.090	<0.001
**ε 4**	-0.393	0.172	0.022
**ε 4/4**	-0.497	0.497	0.317

Sensitivity analysis for unmeasured odor identification at enrollment

Let X_e, denote the vector of observed covariates and observed non-olfactory cognitive scores at enrollment, W denote the probability of being observed at wave three, and let U_e denote the unmeasured odor identification score at enrollment. We further let X_3 denote the measured confounders and cognitive scores at the third wave, and similarly let U_3 be the measured odor identification score at the third wave. Let Z denote the vector of APOE-group indicator-variables.

Following McCandless et al. (1), we use the factorization

$$P(W, U_e \mid Z, X_e) = P(W \mid Z, U_e, X_e) P(U_e \mid Z, X_e)$$

and model the confounding effect of U using a logistic regression for the probability of being observed at the third test wave:

$$logit[P(W = 1|Z, U_e, X_e)] = \beta_0 + \beta_1 X_e + \lambda U_e + \alpha Z$$

and a linear regression model for the unobserved odor identification score at enrollment

$$E(U_e|Z, X_e) = \gamma_0 + \gamma_1 X_e + \omega Z$$

As with other models for unmeasured confounding, the model is non-identifiable and the parameters γ_0, γ_1, ω and λ, reflects assumptions about the confounding effect of U_e. We first assume $(U_e|Z, X_e) = (U_3|Z, X_3)$ in distribution, which reflects as missing at random assumption. Hence, the observed data at the third wave can be used to estimate γ_0, γ_1, and ω. Then we can plug in Z and X_e to predict $E(U_e|Z, X_e)$. As a third step, simulation proceeds as follows:

1. Sample U_e^* from $N(\bar{U}_e, \sigma_{\bar{U}_e})$
2. Fit the following logistic regression model $logit[P(W = 1|Z, U_e^*, X_e)] = \beta_0 + \beta_1 X_e + \lambda U_e^* + \alpha Z$, where λ is either estimated using glm, alternative 1, or set to a fixed value, alternative 2. For the latter, setting λ equal 0.4, reflecting a moderate effect (a stronger effect than using the glm estimate), which is comparable to the parameter estimates of the other non-olfactory cognitive tasks (see Table S1 and Table S2). Noting that U_e^* as well as the other cognitive tasks are first standardized using the z-transformation for comparability.
3. Estimate the longitudinal models using the simulated weights from step 4.
4. Repeat step 1 – 3 K times.

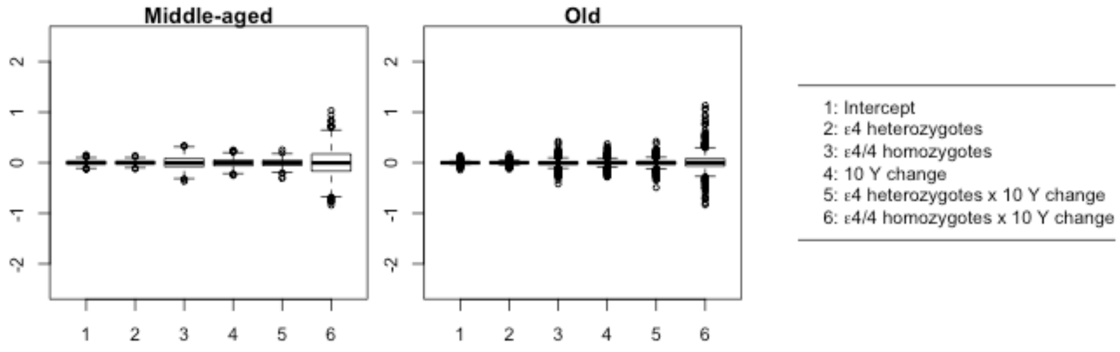

Fig S1: Results from the sensitivity analyses for Middle-aged, first column, and Old participants, second column. Showing boxplots of differences in parameter estimates from the mixed models of Odor identification decline, shown in Table 2 and Table 3 of the main document, and estimates from corresponding mixed models when a simulated odor identification score is included in the IPW models (as described in the simulation procedure above using a moderate effect size (alternative 2)).

References

1. McCandless, L. C., Gustafson, P., & Levy, A. (2007). Bayesian sensitivity analysis for unmeasured confounding in observational studies. *Statistics in medicine*, *26*(11), 2331-2347.
